# Characteristics of Civil Defense Search and Rescue Units, Turkey, 2008-2009

**DOI:** 10.5812/kowsar.20741804.2237

**Published:** 2011-09-15

**Authors:** S Aydogdu, K H Altintas

**Affiliations:** 1 Department of Public Health, Institute of Health Sciences, Hacettepe University, Sihhiye, 06100, Ankara, Turkey

**Keywords:** Civil Defense, Disasters, Emergencies, Evaluation, Search and Rescue, Turkey

## Abstract

**Background:**

Search and rescue (SAR) is a component of emergency and disaster response. SAR teams are limited in number; thus, collecting information on their characteristics may facilitate the establishment of mutual agreement protocols between countries. The objective of this study was to evaluate the characteristics of the Turkish Civil Defense SAR Units.

**Methods:**

This descriptive study was conducted in 11 provinces of Turkey from July 2008 to October 2009. Interviews, observations and records were used to gather data, and descriptive statistics are presented. To evaluate the adequacy of personnel and equipment, a Likert-type scoring system was used (0-4 points).

**Results:**

The size and population density of regions served by SAR Units varied. The mean duration of ground transportation from SAR Unit bases to the furthest provinces in their regions was 4.0±1.2 hours. The mean gathering and loading times were 70.5±42.3 and 48.6±18.0 minutes, respectively. The total employment ratio was 55.6%. The surface and underwater rescue section showed the highest functional sufficiency (3.3±0.7). The mean value for adequacy of SAR equipment was 2.6. Deficiencies were identified in periodic medical check-ups, preventive health measures and after-mission medical examinations for the personnel.

**Conclusion:**

There is a need for standardization and improvement in various characteristics of SAR Units.

## Introduction

**Fig. 1:**
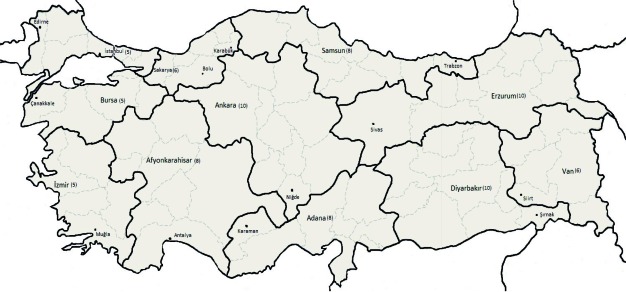
Turkish Civil Defense Search and Rescue units, their regions, the number of provinces in the regions (number in brackets), and the furthest provinces (smaller font) in the regions (Turkey, 2008-2009).

Search and rescue (SAR) is a component of emergency and disaster response. It aims to locate and save people who are in jeopardy and in difficult terrains. First, the victims’ search area is identified, and then the searching operation begins to find the exact site of the victims. Finally, the victims are rescued or their bodies are discovered and transported to a safe area. Lastly, there is a critical phase where the operation is evaluated to determine what could have been done more efficiently.1 Urban SAR refers to SAR conducted in a city. It is the process of locating, extricating and immediately treating victims trapped in collapsed structures. It is generally done after earthquakes or due to other causes leading to building collapse such as explosions.[1][2][3]In Turkey, there are governmental and nongovernmental SAR organizations. Among these, Civil Defense SAR units have a leading and major role. There are 11 Civil Defense SAR units in Turkey. [4]The Ankara SAR Unit has been in operation since August 25, 1986.[5] Istanbul and Erzurum SAR units were established in 1996. Eight SAR units have been functional since January 2001. Figure 1 illustrates the 11 SAR units, their regions and number of provinces, and the furthest province in these regions. As earthquake disasters are frequent in Turkey[6] and a public health concern, these units primarily undertake urban SAR activities.

It is essential for disaster response medical personnel and institutions to have information regarding SAR units. The SAR teams are limited in number;thus, gathering of information on their characteristics and operation may facilitate the establishment of mutual agreement protocols between countries. With these factors in mind, the objective of this study was to evaluate the characteristics of Civil Defense SAR units in Turkey.

## Materials and Methods

This descriptive study was conducted in the Civil Defense SAR units of Adana, Afyonkarahisar, Ankara, Bursa, Diyarbakir, Erzurum, Istanbul, Izmir, Sakarya, Samsun, and Van. The authors developed a general evaluation form that consisted a questionnaire used during interviews with the director of each SAR Unit or with an administrative personnel assigned by the director. The researchers also conducted observations in each unit and used the records/inventories available in the units to transfer data to the evaluation forms. The form included 26 questions in 8 sections. The information regarding size and population data of the SAR Unit regions was taken from the database of the Turkish Statistical Institute and an address-based population registration system. Thirty-five variables regarding the characteristics of SAR units were studied. The study was conducted from July 21, 2008 to October 22, 2009. The authors affirm that our study complies with the Declaration of Helsinki. The locally appointed ethics committee approved the research protocol, and informed consent was obtained from the subjects.

The data were analyzed using SPSS statistical package (Version 17.0, Chicago, IL, USA). The descriptive statistics were presented as frequency and percentage distributions. Mean, standard deviation, minimum-maximum, and median values were given. To evaluate the adequacy of the personnel and equipment, a Likert-type scoring system was used (no idea=0, definitely insufficient=1, insufficient=2, sufficient=3, definitely sufficient=4 points). The local Ethics Committee of Hacettepe University Medical Research (June 6, 2008, LUT 08/27) and the General Directorate of Civil Defense (July 8, 2008, B050SSG0507000-449-3975) approved the study. No financial support was received for the study.

## Results

Afyonkarahisar SAR Unit had the largest (120186 km2, 15.5%) and Sakarya SAR Unit the smallest (25218 km2, 3.3%) region geographically. The mean duration of ground transportation from SAR Unit bases to the furthest provinces in their regions was 4.0±1.2 (min-max=3-7, median=4) hours (Table 1).

**Table 1:** Some characteristics of Civil Defense Search and Rescue units regarding various areas and distances, 2009, Turkey

**Units/Regions**	**Area of the Region **^a^		**The travel time to the furthestprovince in the regionvia ground transport undernormal conditions**		**The total open area size covered by the Unit**		**The total closed area size covered by the Unit**	
	**km^2^**	**%**	**Time (hour)**	**The Furthest Province**	**m^2^**	**%**	**m^2^**	**%**
Afyonkarahisar	120186	15.5	5	Antalya	144676	19.5	10,399	8.3
Ankara	107105	13.9	4	Nigde	61915	8.3	20,000	16.0
Erzurum	105513	13.7	7	Sivas	67214	9.0	1,786	1.4
Diyarbakir	97389	12.7	4	Sirnak	54100	7.3	6,500	5.2
Adana	69790	9.1	6	Karaman	51130	6.9	10,455	8.4
Samsun	61124	7.9	4	Trabzon	80000	10.8	20,000	16.0
Van	58501	7.6	4	Siirt	34441	4.6	19,450	15.7
Izmir	57502	7.5	4	Mugla	37900	5.1	20,000	16.0
Bursa	39803	5.2	4	Canakkale	57786	7.8	7,675	6.1
Istanbul	27473	3.6	3	Edirne	66787	9.0	2,213	1.8
Sakarya	25218	3.3	3	Karabuk	86948	11.7	6,392	5.1
Total	769604	100.0	-	-	742897	100.0	124,870	100.0

^a^ The information regarding the area of the regions was derived from the sum of the surface areas of the provinces included in the regions.[7]

The Istanbul region had a population of 15,949,518 people (22% of Turkey’s population) and a population density of 580.5 people/km2. The Erzurum region had the lowest population density (27.6 people/km2). The Ankara SAR Unit had the highest employment ratio (111 persons, 66.9%), whereas the Van SAR Unit had the lowest (55 persons, 43.3%). The total employment ratio of SAR Units was 55.6% (Table 2). Only a few units had specialized personnel such as physician (2 units), veterinarian (1 unit), social worker (1 unit), and engineer (2 units), with only one present in a specific unit. The director’s position was vacant in the Erzurum and Van SAR units at the time of the study. None of the units had a psychologist or nurse. Each SAR Unit personnel was responsible for approximately 87,213.1 individuals over a 925 km2 service area. These figures varied in each SAR Unit (Table 3).

**Table 2:** Some characteristics of Civil Defense Search and Rescue units regarding population, cadres and employment, 2009, Turkey^a^ [7].

**Units**	**Population in 2000**		**Population in 2009**		**Difference**	**Population density in 2000**	**Population density in 2009**	**Cadres**	**Employed**	**Employment ratio**
**Regions**	**s**	%	**s**	%	**%**	**People/km^2^**	**People/km^2^**	**s**	**s**	**%**
Istanbul	12579478	18.6	15949518	22.0	+3.4	457.9	580.5	146	81	55.5
Adana	7858953	11.6	8668473	11.9	+0.3	112.6	124.2	135	78	57.8
Ankara	8309457	12.3	8574551	11.8	-0.5	77.6	80.1	166	111	66.9
Izmir	7147149	10.5	7908163	10.9	+0.4	124.3	137.5	132	79	59.8
Diyarbakir	6715567	9.9	7009854	9.7	-0.2	68.9	71.9	126	64	50.8
Afyonkarahisar	7180042	10.6	6949167	9.6	-1.0	59.7	57.8	128	70	54.7
Samsun	5.390.166	7.9	4670234	6.5	-1.4	88.2	76.4	135	74	54.8
Bursa	4029381	5.9	4573057	6.3	+0.4	101.2	114.9	127	67	52.8
Erzurum	3478910	5.1	2909868	4.0	-1.1	32.9	27.6	141	81	57.4
Van	2748857	4.1	2853331	3.9	-0.2	46.9	48.7	127	55	43.3
Sakarya	2365967	3.5	2495096	3.4	-0.1	93.8	98.9	134	72	53.7
Total	67803927	100.0	72561312	100.0	-	88.1	94.3	1497	832	55.6

^a^ The database of the address-based population registration system of the Turkish Statistical Institute was used.

**Table 3:** The ratio of SAR personnel and some characteristics regarding Civil Defense Search and Rescue units, 2009, Turkey^a^.

**Units **	**Number of SAR personnel per 100,000 population in 2000**		**Number of SAR personnel per 100,000 population in 2009**		**Surface area (km^2^) per one SAR personnel**		**Number of population per one SAR personnel in 2000**		**Number of population per one SAR personnel in 2009**	
**Regions**	**Cadre**	**Employed**	**Cadre**	**Employed**	**Cadre**	**Employed**	**Cadre**	**Employed**	**Cadre**	**Employed**
Istanbul	1.2	0.6	0.9	0.5	188.2	339.2	86160.8	155302.2	09243.3	196907.6
Adana	1.7	1.0	1.6	0.9	517.0	894.7	58214.5	100755.8	64210.9	111134.3
Ankara	2.0	1.3	1.9	1.3	645.2	964.9	50057.0	74860.0	51653.9	77248.2
Izmir	1.8	1.1	1.7	1.0	435.6	727.9	54145.1	90470.2	59910.3	100103.3
Diyarbakir	1.9	1.0	1.8	0.9	772.9	1521.7	53298.2	104930.1	55633.8	109529.0
Afyonkarahisar	1.8	1.0	1.8	1.0	939.0	1716.9	56094.1	102572.0	54290.4	99273.8
Samsun	2.5	1.4	2.9	1.6	452.8	826.0	39927.2	72840.1	34594.3	63111.3
Bursa	3.2	1.7	2.7	1.5	313.4	594.1	31727.4	60140.0	36008.3	68254.6
Erzurum	4.1	2.3	4.8	2.8	748.3	1302.6	24673.1	42949.5	20637.4	35924.3
Van	4.6	2.0	4.5	1.9	460.6	1063.7	21644.5	49979.2	22467.2	51878.7
Sakarya	5.7	3.0	5.4	2.9	188.2	350.3	17656.5	32860.7	18620.1	34654.1
Total (Turkey)	2.2	1.2	2.1	1.1	514.1	925	45293.2	81.495.1	48471.2	87213.1

^a^ Note: The data presented in Table 1 and 2 were used in the analysis.

The surface and underwater rescue section was determined to have the highest level of functional sufficiency (3.3±0.7). First aid, security and general SAR sections received 1 point as a minimum score. The mean and median values for the sufficiency of SAR equipment were 2.6 and 3.0 points, respectively. With regards to the sufficiency of equipment, NBC (nuclear, biological and chemical) decontamination, first aid, transportation, and security sections received 1 point as a minimum score (Table 4). Some equipment, such as oxy-fuel cutting torch, had been never used.

**Table 4:** The sufficiency of Civil Defense Search and Rescue units regarding personnel and equipment, 2009, Turkey^a^

**Divisions of the Units**	**Sufficiency of the personnel**			**Sufficiency of the equipment**		
	**Mean±Standard deviation**	**Min.-max.**	**Median**	**Mean±Standard deviation**	**Min.-max.**	**Median**
Surface and underwater SAR	3.3±0.7	2-4	3	3.1±0.3	3-4	3
Mountain-avalanche SAR	2.8±1,1	2-4	3	3.2±0.4	3-4	3
General SAR	2.7±0,8	1-4	3	3.1±0.3	3-4	3
Earthquake SAR	2.7±1.0	2-4	3	3.0±0.5	2-4	3
Education and public relations	2.7±1.0	2-4	3	2.9±0.3	2-3	3
Social assistance	2.5±1.2	3-3	3	3.0±0.0	3-3	3
Nuclear, biological, chemical (NBC) decontamination	2.5±1.3	2-4	3	2.6±0.9	1-4	3
Communication	2.5±1.3	2-4	3	2.7±0.9	3-3	3
Canine SAR	2.4±1.3	2-4	3	3.0±0.5	2-4	3
Transportation and technical affairs	2.4±1.3	2-4	3	2.7±0.7	1-3	3
Logistics and equipment	2.3±1.2	2-3	3	2.9±0.3	2-3	3
Personnel and management	2.3±1.2	2-3	3	2.8±0.4	2-3	3
First aid and ambulance	2.1±1.2	1-3	3	2.6±0.8	1-3	3
Security	1.8±1.4	1-4	2	2.6±0.8	1-3	3

^a^ Divisions were evaluated as: No idea=0, definitely insufficient=1, insufficient=2, sufficient=3, definitely sufficient=4 points.

On the average, there were 3±1 (29 in total) SAR canines in the units. SAR canines were trained mostly by volunteers and were then transferred to the units. All the SAR units had the ability to provide canine, technical, underwater, fire, flood, collapsed building, earthquake, and mountain SAR services, and monitoring and decontamination of nuclear and chemical materials. Ten SAR units had the ability to provide monitoring and decontamination of biological materials, food service (distribution of food), first aid, and fire extinction. Nine SAR units had the ability to provide shelter (setting up tents), and 7 SAR units had the ability to remove debris, a task requiring heavy equipment. The SAR units also trained volunteers. As of December 31, 2007, 153,841 volunteers were trained.

All the SAR units had common disaster drills with provincial civil defense directorates, 112 emergency medical services, and national medical rescue teams (UMKE), the army, and the directorate of security. Common disaster drills were also organized with the fire brigade (10 SAR units), Turkish Red Crescent (9 SAR units), non-governmental SAR organizations (7 SAR units), neighborhood disaster volunteers (MAG) (4 SAR units), and the Turkish Society of Radio Amateurs (TRAC) (1 SAR Unit).

The personnel of 5 SAR units underwent periodic medical check-ups. All the personnel of SAR units had received tetanus and hepatitis vaccinations. In 10 SAR units, no preventive health measures were taken for the personnel before their deployment for a mission in a foreign country (Table 5). Public shelters were not ready for use in any of the regions. While the facilities/buildings of 10 SAR units were certified with respect to earthquake resistance, some of the buildings of the Izmir SAR Unit were uncertified.

**Table 5:** Disaster preparedness characteristics of Civil Defense Search and Rescue units, 2009, Turkey.

**Disaster preparedness characteristics (n=11)**	**n**
The personnel are vaccinated with regard to their duties.	11
The unit has a disaster plan.	11
There is a deployment plan for search and rescue tasks.	11
There is a directive for the functioning of the communication center.	11
The communication center functions 24 hours a day.	11
Contact information of the personnel is available at the communication center.	11
In case of a disaster, food packets are available for the personnel to suffice for a minimum of 3 days.	11
The vehicles and equipment that are used in search and rescue are ready to function and periodic maintenance is provided.	11
The deployment plan has been approved by the general directorate.	10
The search and rescue vehicles and equipment are continuously renewed in accordance with international standards.	10
There is a detailed map of the region.	9
The communication center does not release information to the media.	8
The disaster plan of the unit is updated.	8
Drills are exercised based on the disaster plan of the unit.	8
In need of aerial deployment, the appropriate authority for request of air vehicles is known.	7
Vital facilities, resources, main transportation roads, gathering areas, etc. are designated on the map of the region (n=9).	7
The environmental risks that may lead to a disaster in the region are determined.	7
In need of maritime deployment, the appropriate authority for request of maritime vehicles is known.	6
The personnel undergo periodic check-ups with regard to their duty.	5
The alert and warning systems in the region are functioning.	3
Personal preventive health measures are taken against the country specific health problems before deployment of personnel to that country.	1

The mean gathering and loading times of SAR units were 70.5±42.3 (min-max=40-180, median=60) and 48.6±18.0 (min-max=30-90, median=45) minutes, respectively. According to 2 SAR units, when multiple SAR units were deployed to a disaster site, the most experienced director should lead the overall operation. However, other SAR units stated that the director of the SAR Unit in charge of the affected region should serve as leader (Table 6). After SAR missions, the personnel underwent routine medical check-ups in only 4 SAR units (Table 7).

**Table 6:** Some activities of Civil Defense Search and Rescue units during disaster response, 2009, Turkey.

**Activities during disaster response (n=11)**	**n**
The personnel are briefed before the mission (emergency situation, cultural characteristics of the victims, weather condition, security issues and emergency evacuation).	11
The personnel work in shifts in the affected area.	11
Cultural, moral and legal issues of the affected population are taken into account.	11
Personal protective equipment is used.	11
Risks in the disaster site are analyzed (secondary hazards like downed power lines, gas leakage, explosive materials, etc.).	11
Security of the personnel is established at the disaster site.	11
The personnel do not depend on local food stocks and have enough food for a minimum of 3 days.	11
At the disaster site, repairs are done for simple search and rescue equipment failures.	11
GPS is used.	11
Hazardous materials are detected (nuclear, biologic, chemical and radioactive).	11
At the incident site, the personnel answer questions from the media.	11
There is a standard procedure for marking the buildings.	10
The Unit can provide life support to its personnel, search and rescue dogs, and victims.	10
Hygienic shelters are provided to the personnel.	9
A system is established to continuously gather and analyze information regarding disasters.	6
A phonetic alphabet is used in radio communications.	6
During missions in a foreign country, the unit’s own communication system is established.	6
When more than one unit is deployed to a disaster site, the most experienced unit director assumes the lead.	2

**Table 7:** Post-disaster response activities of Civil Defense Search and Rescue units 2009, Turkey.

**Post-disaster response activities (n=11)**	**n **
After the mission, search and rescue materials undergo control, counting and maintenance.	11
End duty reports are sent to higher authorities.	11
At the end of the duty, verbal feedback is taken from the personnel.	10
At the end of the duty, written feedback is taken from the personnel.	10
At the end of the duty, the personnel are given a leave for rest.	10
After the mission, the personnel receive check-ups.	4

The major problems as stated by the SAR units were related with inadequate personal rights (overtime pay, compensation for risk and burnout, ration allowance, field pay, etc.: 11 SAR units), cadres (insufficient personnel: 7 SAR units), vehicles (inappropriate for the region and mission: 6 SAR units), facilities (2 SAR units), coordination with other organizations (2 SAR units), E-class driving license (lack of enough personnel with E-class driving licence: 1 SAR Unit), vacation (1 SAR Unit), country-wide radio relay (1 SAR Unit), personnel radios (inadequate supply of radios for all personnel: 1 SAR Unit), and standard inflatable tents (1 SAR Unit).

The SAR units were queried regarding any recommendations and they may improve their capabilities. Five SAR units emphasized the significance of inter-provincial and inter-regional disaster field exercises and professional training exercises. Four SAR units stated that responding to national and international disasters would increase their experience.

## Discussion

As the employment ratio of SAR units was 55.6% and 7 units complained about the lack of personnel, understaffing was considered as an important obstacle to ensuring successful and sufficient SAR operations. In 2001, when 8 new SAR units were established, the personnel recruited were distributed among them to render the units functional as early as possible. In such a short time, it was not possible to recruit as many personnel to fill all the positions available in these 8 units. The economic crisis also likely contributed to the under-assignment. The authorities further explained that if all the positions were filled simultaneously, a serious experience loss would occur at the time of retirement, because nearly all the members of a Unit would retire over a relatively short period. With this in mind, the authorities planned to fill the positions slowly over time.

For the functional sufficiency of the personnel, some sections received the minimum 1 point. This low score can probably be attributed to the lack of personnel rather than to professional insufficiencies. The lack of personnel was more prominent in SAR (SAR technician, engineer), first aid (physician, emergency medical technician, etc.) and security (security personnel) sections. With few exceptions, there were no physicians, nurses, psychologists or veterinarians in the SAR units. It seemed that it would not be possible to assign these health professionals to the available posts in the SAR units in the near future, which could have a severe negative impact on the health component of SAR. A possible solution to this problem might be coupling the responses of SAR units with national medical rescue teams; in fact, this is a common practice today. The national medical rescue teams are composed of volunteer health personnel with special training. They work in coordination with SAR personnel and provide medical assistance. Emergency Medical Service (EMS) personnel, specifically emergency medical technicians and paramedics, could participate in the SAR Unit activities as well. However, this requires the training of EMS (ambulance) personnel in SAR operations. Another issue was the lack of drivers with the appropriate driving license. To solve this problem, SAR technicians with an appropriate license were ordered to drive the vehicles when moving to a disaster site. However, this will be an additional burden on the SAR technicians, tiring them before their arrival to the site. A better solution would be the assignment of logistics personnel to operate the vehicles. The international organizations use this system as well, so the personnel and logistics demand are minimized.[7][8]

When an earthquake hits an urban area, the first 24 hours are vital for SAR activities.[2] Thereafter, the probability of rescuing live victims who are trapped declines rapidly. There is thus a race against time, and SAR activities should be continuous, which necessitates working in shifts. For this, the available SAR personnel should be divided into groups, which in Turkey’s situation, will further decrease the number of personnel. As a result, a SAR Unit lacking an adequate number of personnel will be further handicapped in its functions. On the contrary, overassignment can lead to problems as well. The operation area will be more crowded than necessary, which can hinder the SAR activity. Coordinating the crowded personnel, the required logistics, and maintaining a silent environment during sensitive acoustic listening, etc. can be cited as some of the potential problems. Thus, the number of personnel in SAR units should be optimal. The Federal Emergency Management Agency (FEMA) determined the optimal size of a SAR team as 70 persons.[8] In Turkey, the criteria used to determine the number of personnel in SAR Unit were not clear. The calculation of the Unit size seemed to be dependent on geography, population density and risks, but the specific criteria were not defined in the related legislation.[4]

The mean gathering and loading times of SAR units were 70.5 and 48.6 minutes, respectively. Under normal conditions, the mean duration of ground transportation from SAR Unit bases to the furthest provinces in their regions was 4.0 hours. When gathering, loading and transportation times were added, it took approximately 6 hours at most to arrive at a disaster site in the SAR Unit’s region. In the United States, FEMA determined this time as 24 hours.[8] Thus, 6 hours can be considered an acceptable time in a country like Turkey.

The Istanbul region has the highest population density among the regions, which is 6.2 times greater than the population density of Turkey. The Istanbul region also has a high earthquake risk. Thus, in the event of an earthquake, there might be a high death toll and severe damage to the building stock.[9] In order to mitigate an earthquake disaster, a project entitled “Istanbul Seismic Risk Mitigation and Emergency Preparedness Project (ISMEP)” has been ongoing since February 3, 2006.[10] This project was supported by the World Bank. However, due to various factors (population density, fragile building storage, etc.), these efforts (primary prevention) might not be sufficient and there might be a need for extensive SAR activity (secondary prevention).[11] In the case of an earthquake disaster in the Istanbul region, the Sakarya SAR Unit will be the first back-up Unit to the Istanbul SAR Unit. Further, in every province, an additional 10-30 SAR personnel are available who are not personnel of SAR units.[4] Thus, they could also contribute to the SAR efforts in addition to other governmental and nongovernmental SAR teams. The SAR canines would be very useful in such an event, but the number of SAR canines is low and the methods of their training require further investigation.

The SAR materials, equipment and vehicles varied in type, model, size, power, etc. and possessed different properties. Furthermore, some technical devices and equipment were designated differently by name. It was therefore difficult to evaluate the equipment and devices utilized in the SAR units. It was also not possible to obtain a standard list of SAR materials, equipment and devices present in the SAR units. There were also modernization problems regarding materials, equipment and vehicles. As the SAR units may play a role in international SAR as well, the International Search and Rescue Advisory Group (INSARAG) standards should be accepted and implemented.[12] The efforts to have an INSARAG certificate will aid in improving both sufficiency and professionalism.

The SAR units and other governmental organizations in the corresponding regions were reported as having common disaster drills. To improve the sufficiency and skill of personnel, all the available equipment and devices should be utilized in these exercises. The vehicles of SAR units are among the important factors affecting the capacity of the Units. During the deployment of SAR units, if any vehicular malfunction occurs, the SAR response will be delayed. Therefore, maintenance of the vehicles is of prime importance. The vehicles should operate at a sufficient speed to ensure that the convoy can arrive at its destination in as short a time as possible. The vehicles should be operable in the geography and climate of the regions, and should be able to transport at least 5 personnel and the equipment.

Only the personnel of 5 SAR units received periodic medical check-ups. Normally, when a rescuer feels ill, he/she can be referred to a family physician or hospital. However, for early diagnosis and treatment, periodic medical check-ups are vital. The SAR units should establish ties with regional, nearby health facilities for periodic medical check-ups. As the international SAR assignments were infrequent, the arrangements for preventive measures against exotic diseases were not sufficient in the SAR units.

All SAR units had disaster plans; however, only 7 SAR units evaluated the hazards in their regions and updated their plans. All these hazards, vital facilities, resources, and public shelters should be mapped. Although public shelters and alerting-warning systems are the responsibility of the provincial authorities, SAR units should inspect them and ensure their preparedness. All these instruments will enable SAR units to function more rapidly, efficiently and in coordination. They will also facilitate the efficiency of the SAR Unit of the region in coordinating the assisting SAR units and teams that are unfamiliar with the region.

When multiple SAR units are operating in a region for the same event, which director should assume the role of coordinator remains unclear. However, article 12 of the legislation regarding the foundation, duties, working principles, and procedures of the Civil Defense SAR units and teams explains this issue clearly.[4] According to the legislation, when multiple units are operating in a region for the same event, the leader should be the director of the region’s SAR Unit. If the regional SAR Unit’s Director is not available, the most experienced SAR Unit Director should take the lead.

After SAR missions, the personnel underwent routine medical examinations in only 4 SAR units. Some obscure injuries might go undetected, and there is potential risk of contracting undiagnosed contagious diseases.[2] Some health problems that might be treated with simple interventions, when left untreated, can lead to serious health problems for the personnel. Also, in the case of contagious diseases, there is a possible risk of resultant epidemics as well. Thus, a group or perhaps all of the personnel might become incapacitated. The mental health of the personnel should be evaluated after every mission.[13] The best means of solving these health issues of the SAR personnel may be through assigning a permanent health personnel to the Unit or by making the necessary arrangements with the nearest regional health facility. The major problems as stated by the SAR units were related with insufficiencies of personal rights, which can be addressed by passing appropriate legislation.

The descriptive nature of the study and our inability to observe the SAR units while on active duty can be mentioned as two limitations of the study. The strength of the study is that it is the first such detailed study on the characteristics of SAR units in Turkey.

In conclusion, there is clearly a need for standardization and improvement in various characteristics of the SAR units. When this is done, SAR units will be a valuable asset in both national and international disaster responses.
